# Progesterone Is Associated With Increased Vasohibin‐2 Expression, Tubulin Detyrosination, and Paclitaxel Sensitivity in PR‐Negative Ovarian Cancer Cells

**DOI:** 10.1002/cnr2.70681

**Published:** 2026-09-06

**Authors:** Takahiro Koyanagi, Yasushi Saga, Yoshifumi Takahashi, Kohei Tamura, Eri Suizu, Kazuki Yamamoto, Akiyo Taneichi, Yuji Takei, Hiroaki Mizukami, Hiroyuki Fujiwara

**Affiliations:** ^1^ Department of Obstetrics and Gynecology School of Medicine, Jichi Medical University Shimotsuke Tochigi Japan; ^2^ Division of Genetic Therapeutics Center for Molecular Medicine, Jichi Medical University Shimotsuke Tochigi Japan

**Keywords:** cell cycle, detyrosinated tubulin, ovarian cancer, paclitaxel, progesterone, vasohibin‐2

## Abstract

**Background:**

Progesterone induces rapid cellular responses in progesterone receptor (PR)‐negative ovarian cancer cells that are consistent with non‐genomic progesterone signaling. Vasohibin‐2 (VASH2), originally identified as a pro‐angiogenic factor, has recently been recognized as a tubulin carboxypeptidase involved in microtubule regulation.

**Aims:**

This study investigated the association of progesterone treatment with VASH2‐related molecular changes and paclitaxel sensitivity in ovarian cancer cells.

**Methods:**

Two PR‐negative ovarian cancer cell lines expressing membrane progesterone receptors (mPRs) were treated with progesterone. VASH2 mRNA expression was evaluated by RT‐qPCR, whereas detyrosinated tubulin and cyclin B1 expression were assessed by western blotting. Paclitaxel and gemcitabine sensitivities were determined using WST‐1 cell viability assays.

**Results:**

Progesterone treatment was associated with increased VASH2 mRNA expression, enhanced tubulin detyrosination, cyclin B1 accumulation, and significantly reduced paclitaxel IC_50_ values in both cell lines. In contrast, progesterone had no significant effect on gemcitabine sensitivity.

**Conclusion:**

Progesterone treatment was associated with increased VASH2 expression, enhanced tubulin detyrosination, and increased paclitaxel sensitivity in PR‐negative ovarian cancer cells. These findings provide in vitro evidence supporting further mechanistic and preclinical investigation of progesterone as a potential adjunct to paclitaxel therapy.

**Abbreviations**
Cdk: cyclin‐dependent kinase
FBS: fetal bovine serum
GEM: gemcitabine
mPR: membrane progesterone receptor
mRNA: messenger ribonucleic acid
PR: progesterone receptor
PTX: paclitaxel
PVDF: polyvinylidene fluoride
qPCR: quantitative polymerase chain reaction
RT‐qPCR: reverse transcription‐quantitative polymerase chain reaction
SDS: sodium dodecyl sulfate
TBS‐T: tris‐buffered saline with Tween 20
VASH1: vasohibin‐1
VASH2: vasohibin‐2
VEGF: vascular endothelial growth factor

## 1 Introduction

Ovarian cancer is the sixth leading cause of cancer‐related death among women in the United States. Approximately 21 000 women are newly diagnosed each year, and nearly 13 000 women die from this disease annually [1]. Because early‐stage ovarian cancer is often asymptomatic, more than half of patients are diagnosed at an advanced stage with peritoneal dissemination and malignant ascites. The current standard treatment for advanced ovarian cancer consists of primary cytoreductive surgery, followed by combination chemotherapy with platinum, a taxane, and bevacizumab. Ovarian cancer is relatively sensitive to such chemotherapy, and many patients initially achieve complete or partial remission with this multimodal approach. However, the therapeutic benefit is typically transient, with more than half of patients experiencing recurrence and ultimately succumbing to the disease [2, 3]. Therefore, the development of novel therapeutic strategies remains an urgent clinical need.

The vasohibin family consists of vasohibin‐1 (VASH1) and vasohibin‐2 (VASH2). VASH1 was originally identified as an angiogenesis inhibitor produced by vascular endothelial cells [4]. We previously demonstrated that forced expression of VASH1 suppresses tumor growth and peritoneal dissemination, and prolongs survival in ovarian cancer animal models [5, 6]. VASH2, a homolog of VASH1, was subsequently identified and shown to be predominantly produced by tumor cells, including ovarian cancer cells, and to promote angiogenesis, in contrast to VASH1 [7]. VASH2 is scarcely expressed in normal tissues [8], and VASH2‐knockout mice exhibit no apparent phenotypic abnormalities [9]. These findings suggest that therapeutic strategies targeting VASH2 may be effective while causing minimal adverse effects.

Recent studies have revealed that VASH2 possesses tubulin carboxypeptidase activity, promoting microtubule detyrosination and thereby influencing microtubule regulation in cancer cells [10, 11]. Paclitaxel (PTX), a cornerstone chemotherapeutic agent for ovarian cancer, exerts antitumor effects by inhibiting microtubule dynamics. We recently reported that VASH2‐knockout ovarian cancer cells exhibit reduced microtubule activity and markedly increased sensitivity to PTX [12], indicating that targeting VASH2 may synergize with conventional chemotherapy.

Progesterone is a steroid hormone primarily synthesized by the ovarian corpus luteum. Classically, progesterone binds to nuclear progesterone receptors (PRs), forming a complex that regulates gene transcription through interaction with promoter regions in the nucleus [13]. In oncology, progesterone preparations are currently used to treat endometrial and breast cancers with fewer side effects; however, their precise mechanisms of action remain incompletely understood [14, 15]. More recently, membrane progesterone receptor (mPR) family members have been identified as cell‐surface receptors mediating rapid, non‐genomic progesterone signaling [16]. These mPRs are known to induce rapid cellular responses distinct from those mediated by classical genomic signaling via nuclear PRs [17]. We previously demonstrated that progesterone rapidly induces cell death in ovarian cancer cells through mPR‐mediated non‐genomic mechanisms [18]. Furthermore, we recently reported that progesterone enhances SN38 sensitivity by suppressing topoisomerase I expression and inducing ferroptosis in ovarian cancer cells [19]. SN38, the active metabolite of irinotecan, is used as second‐line chemotherapy for platinum‐ and taxane‐resistant recurrent ovarian cancer [20].

This study investigated the effects of progesterone on VASH2 expression and PTX sensitivity in ovarian cancer cells, aiming to explore a novel therapeutic strategy.

## 2 Methods

### 2.1 Cell Lines and Cell Culture

The human ovarian serous adenocarcinoma cell line TU‐OS‐4 was provided by the establisher [21]. The human ovarian serous adenocarcinoma cell line OVKATE was purchased from the Japanese Collection of Research Bioresources (JCRB, Osaka, Japan). The cell lines were authenticated by an expert before use in the experiments. Mycoplasma contamination was excluded using a polymerase chain reaction (PCR)‐based mycoplasma detection kit (Takara Bio Inc., Tokyo, Japan). In our previous studies, these cells did not express PR but expressed various mPRs [18]. These cells were cultured in phenol red‐containing Dulbecco's Modified Eagle Medium/F12 (DMEM/F12; Thermo Fisher Scientific Inc., Waltham, MA, USA) supplemented with 10% heat‐inactivated fetal bovine serum (FBS; Sigma Aldrich; Merck KGaA, Darmstadt, Germany) and 1% penicillin/streptomycin (Thermo Fisher Scientific Inc.) at 37°C under 5% CO_2_.

### 2.2 Quantitative Reverse Transcription‐Polymerase Chain Reaction (RT‐qPCR)

Tumor cells (2 × 10^5^ cells/well) seeded onto a six‐well plate were exposed to progesterone (FUJIFILM Wako Pure Chemical Co., Osaka, Japan) at a concentration of 400 μM for 30 min. Cellular messenger ribonucleic acid (mRNA) was extracted using an RNeasy Mini Kit (Qiagen, Valencia, CA, USA) according to the manufacturer's instructions. RT‐qPCR was performed using the Thermal Cycler Dice Real Time System II (Takara Bio Inc.) following the manufacturer's instructions. PCR was carried out using 40 cycles of heating at 95°C for 15 s, 58°C for 15 s, and 72°C for 20 s. The mRNA level of VASH2 was determined relative to the fluorescence signal of glyceraldehyde‐3‐phosphate dehydrogenase (GAPDH). Primer sequences are as follows: GAPDH: forward: 5′‐ACCACAGTCCATGCCATCAC‐3′, reverse: 5′‐CATCACGCCACAGTTTCCCG‐3′; VASH2, forward: 5′‐CTACTTAACCAATGGGCAGC‐3′, reverse: 5′‐CTTCATTCTCATGTCCCTGG−3′.

### 2.3 Western Blot Analysis

Tumor cells were seeded onto six‐well plates at 2 × 10^5^ cells/well; after 24 h, cells were exposed to progesterone at a concentration of 400 μM for 30 min. Tumor cells were lysed using lysis buffer (1% NP 40, 150 mM NaCl, 50 mM Tris HCl, pH 8.0), and extracted proteins were mixed with 1% sodium dodecyl sulfate (SDS) sample buffer (10 mM Tris HCl, pH 7.5, 150 mM NaCl, 1% SDS, EDTA free protease inhibitor cocktail [Roche, Basel, Switzerland]), separated by electrophoresis on 10% polyacrylamide gels, and transferred onto polyvinylidene fluoride (PVDF) membranes (Merck KGaA, Darmstadt, Germany). Membranes were incubated at room temperature for 1 h in PVDF Blocking Reagent for Can Get Signal (Toyobo Life Science, Osaka, Japan), washed three times using Tris‐buffered saline containing Tween 20 (TBS‐T), and incubated overnight at room temperature with the following antibodies diluted in Can Get Signal Immunoreaction Enhance Solution 1 (Toyobo Life Science): Anti‐detyrosinated α‐tubulin rabbit polyclonal antibody (cat. no. ab48389; Abcam, Cambridge, UK), anti α‐tubulin mouse monoclonal antibody (cat. no. sc32293; Santa Cruz Biotechnology Inc., Dallas, TX, USA), anti‐cyclin B1 rabbit monoclonal antibody (cat. no. 12231; Cell Signaling Technology Inc., Danvers, MA, USA), and anti α‐actin rabbit polyclonal antibody (cat. no. A2066; Sigma Aldrich; Merck KGaA). After the reaction, membranes were washed three times with TBS‐T and incubated with peroxidase‐labeled anti‐mouse antibody (cat. no. NA9310V; GE Healthcare Japan, Tokyo, Japan) or peroxidase‐labeled anti‐rabbit antibody (cat. no. NA9340V; GE Healthcare Japan) in Can Get Signal Immunoreaction Enhance Solution 2 (Toyobo Life Science) at room temperature for 1 h. Membranes were washed three times with TBS‐T, incubated with enhanced chemiluminescence (ECL) prime western blotting detection reagent (GE Healthcare Japan), and imaged using a cooled charge‐coupled device (CCD) system (LAS 4000 mini; GE Healthcare Japan). Relative protein expression levels were compared with those of α‐tubulin or α‐actin set as 1.0 using ImageJ software (NIH, Bethesda, MD, USA).

### 2.4 Colorimetric Assay

Tumor cells (1000 cells/100 μL/well) were exposed to progesterone at concentrations of 0 or 100 μM for 30 min; and after complete medium removal, cells were exposed to PTX (Sawai Pharmaceutical Co. Ltd., Osaka, Japan) at concentrations of 1–128 nM for 48 h, or exposed to gemcitabine (GEM, Nichi‐Iko Pharmaceutical Co. Ltd., Tokyo, Japan.) at concentrations of 1–256 μM for 48 h. Cell viability was measured using the Premix WST‐1 Cell Proliferation Assay System (Takara Bio Inc.) and expressed as a percentage of that of untreated control cells.

### 2.5 Statistical Analysis

Statistical analysis was performed using EZR software (Saitama Medical Center, Jichi Medical University, Saitama, Japan). Student's *t*‐test was used to compare the two groups. *p*‐values < 0.01 were considered statistically significant.

## 3 Results

### 3.1 VASH2 Expression

**FIGURE 1:**
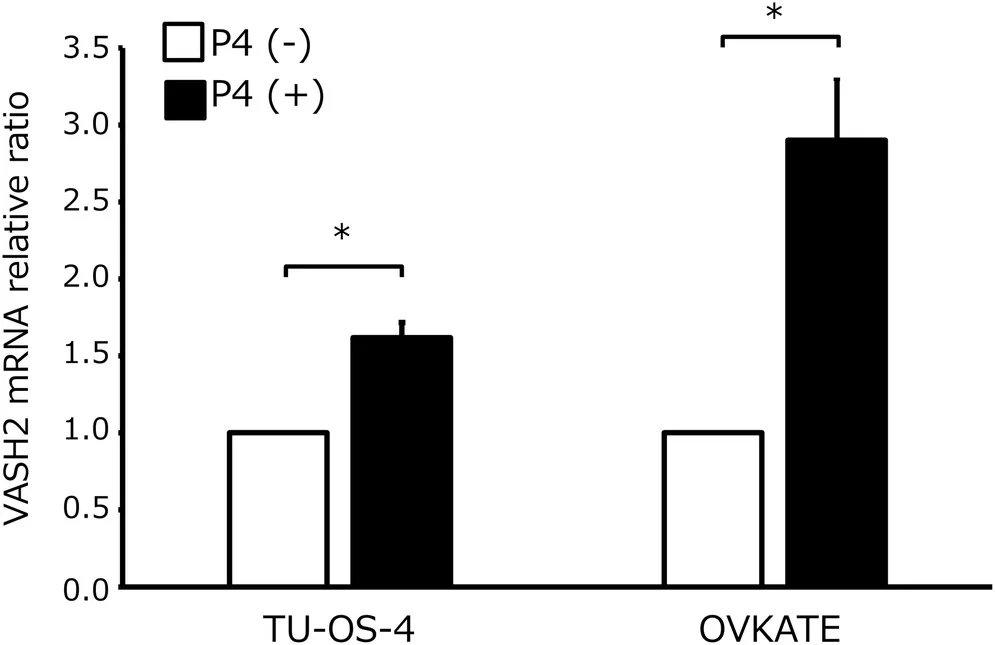
Vasohibin‐2 (VASH2) gene expression and progesterone exposure. Reverse transcription‐quantitative polymerase chain reaction data show that VASH2 expression is significantly higher in ovarian cancer cell lines exposed to progesterone (400 μM, 30 min). Data are shown as the mean and standard deviation (*n* = 3). *p < 0.01 (vs. progesterone‐untreated cells). P4, progesterone; VASH, vasohibin.

We examined the effects of progesterone on VASH2 expression in ovarian cancer cell lines. As shown in Figure 1, progesterone increased VASH2 expression in both TU‐OS‐4 and OVKATE cells. These results suggest that progesterone‐induced VASH2 expression in ovarian cancer cells.

### 3.2 Tubulin Detyrosination

**FIGURE 2:**
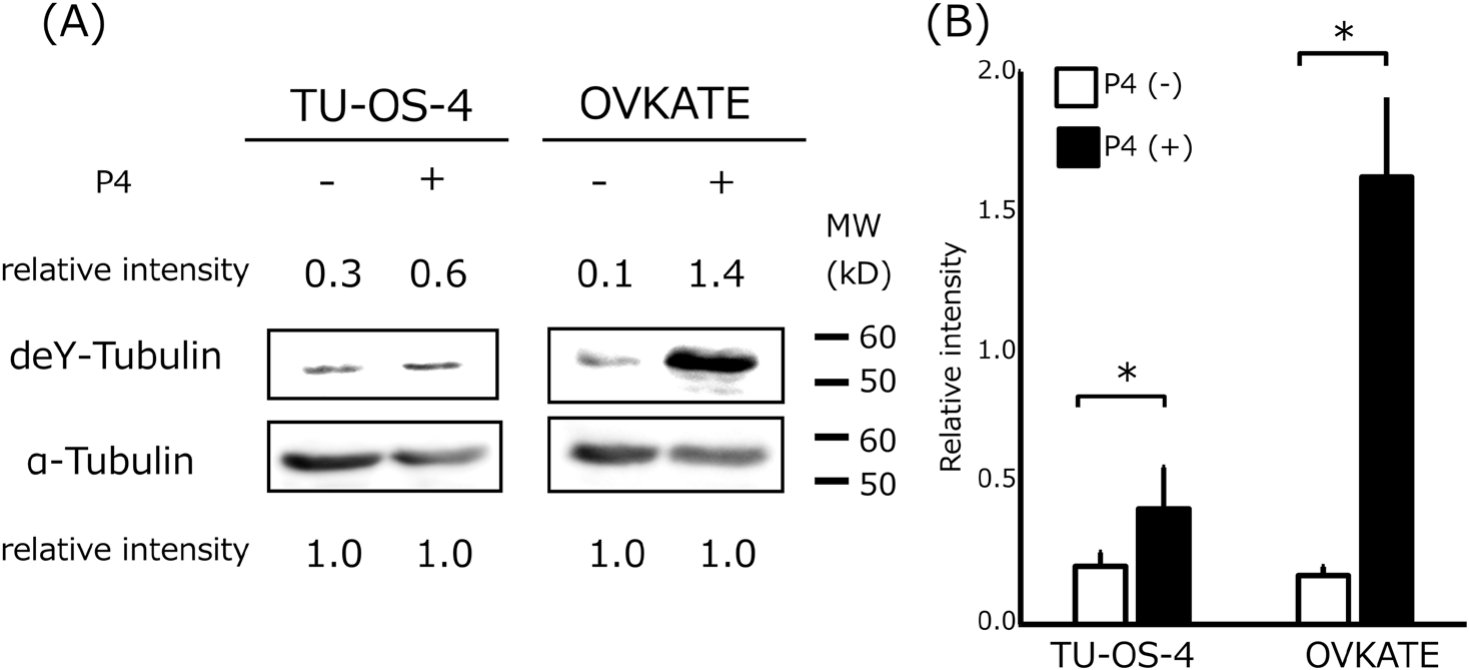
Western blotting of detyrosinated tubulin (deY‐tubulin) in ovarian cancer cell lines with or without progesterone exposure. (A) Progesterone‐untreated cells weakly express detyrosinated tubulin, whereas progesterone‐treated cells show increased detyrosinated tubulin expression. α‐Tubulin in the cell lysate serves as a loading control. Each number indicates the relative protein expression level, with α‐tubulin set at 1.0. (B) Densitometric analysis is presented as a column graph. Data are shown as the mean and standard deviation (*n* = 3). *p < 0.01 (vs. progesterone‐untreated cells). P4, progesterone.

Next, we evaluated the intracellular detyrosinated tubulin status with or without progesterone exposure. Figure 2 shows western blot analysis of detyrosinated tubulin in the ovarian cancer cell lines TU‐OS‐4 or OVKATE. Compared with progesterone‐untreated controls, progesterone‐treated cells showed increased expression of detyrosinated tubulin. These results suggest that progesterone increased intracellular tubulin detyrosination in ovarian cancer cells.

### 3.3 Cyclin B1 Expression

**FIGURE 3:**
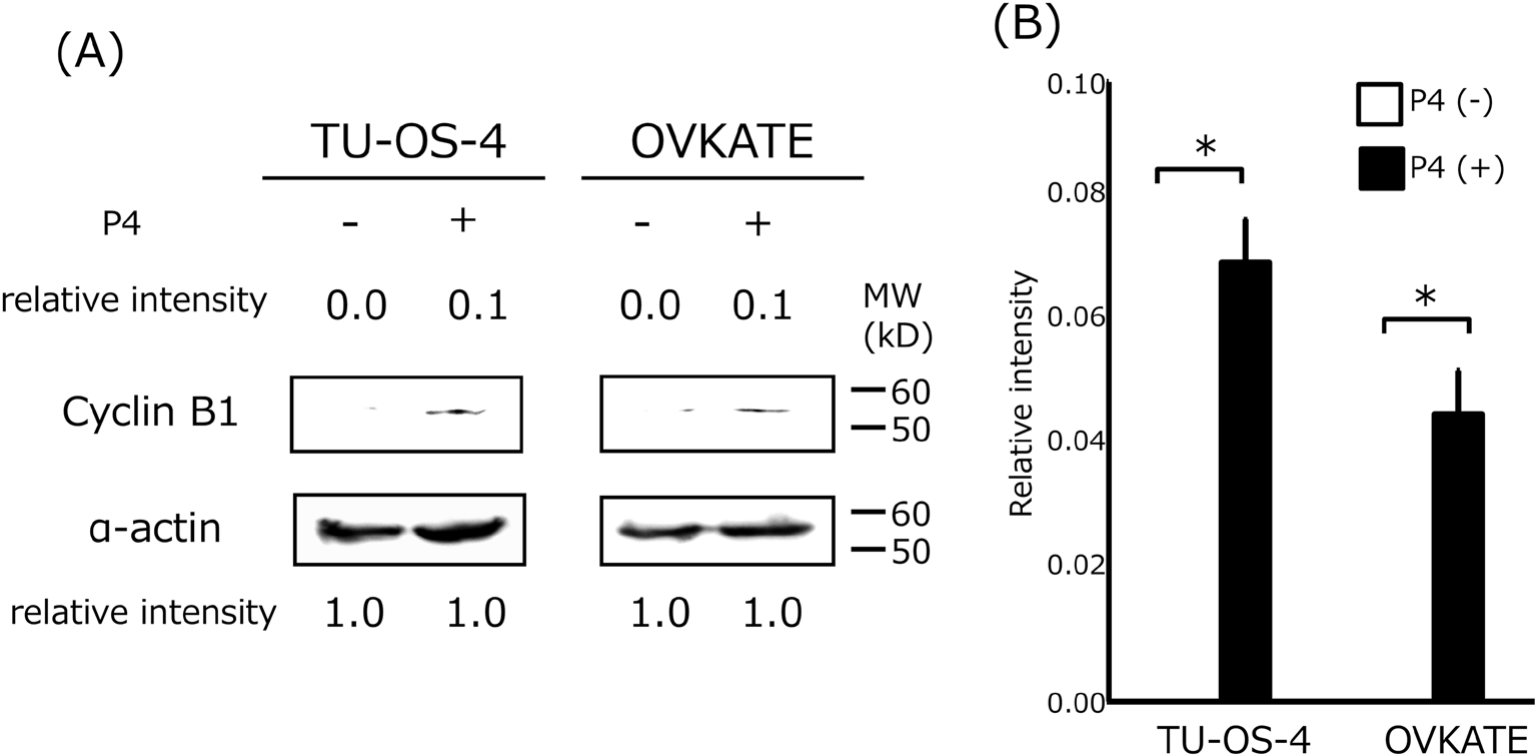
Western blotting of cyclin B1 in ovarian cancer cell lines with or without progesterone exposure. (A) Cyclin B1 expression is weakly detected in progesterone‐treated cells, whereas progesterone‐untreated cells show minimal cyclin B1 expression. α‐Actin in the cell lysate serves as a loading control. Each number indicates the relative protein expression level, with α‐actin set at 1.0. (B) Densitometric analysis is presented as a column graph. Data are shown as the mean and standard deviation (*n* = 3). *p < 0.01 (vs. progesterone‐untreated cells). P4, Progesterone.

Cyclin B1 expression was evaluated by western blot analysis. As shown in Figure 3, cyclin B1 protein expression was detected in progesterone‐treated TU‐OS‐4 and OVKATE cell lines, whereas no cyclin B1 expression was detected in progesterone‐untreated cell lines. These results suggest that progesterone increased cyclin B1 expression in ovarian cancer cells.

### 3.4 Chemosensitivity

**FIGURE 4:**
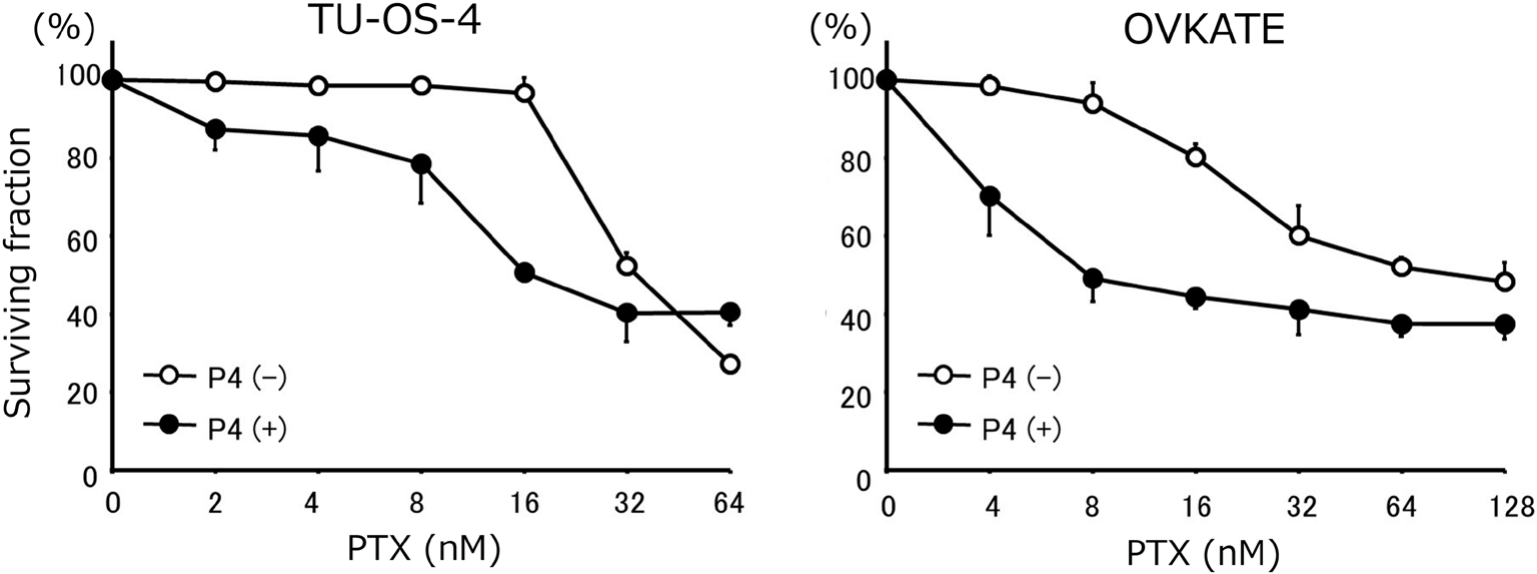
Chemosensitivity to paclitaxel (PTX). The IC_50_ for PTX in TU‐OS‐4 cells is 19.8 ± 6.0 nM with progesterone versus 35.3 ± 3.4 nM without progesterone (1.8‐fold higher sensitivity, *p* < 0.01). The IC_50_ for PTX in OVKATE cells is 9.5 ± 32.6 nM with progesterone versus 128.0 ± 64.0 nM without progesterone (13.5‐fold higher sensitivity, *p* < 0.01). Data are presented as the mean and standard deviation (*n* = 3). P4, progesterone; PTX, paclitaxel.

**FIGURE 5:**
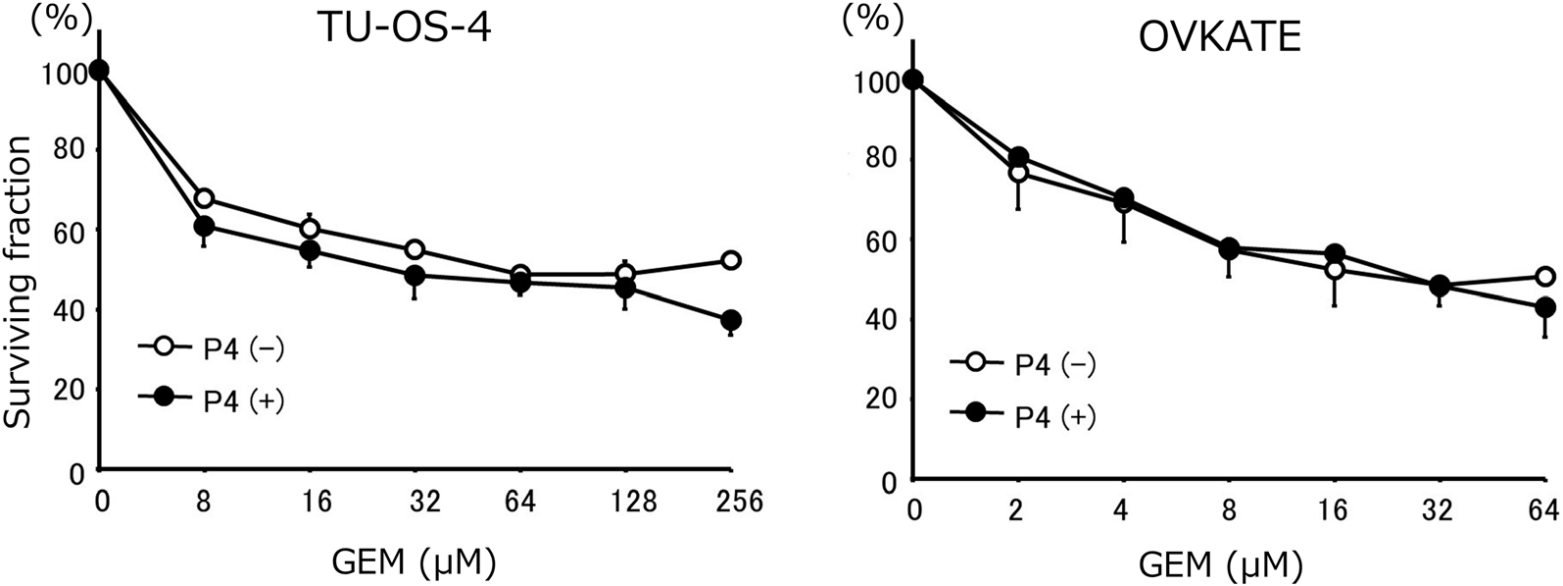
Chemosensitivity to gemcitabine (GEM). The IC₅₀ for GEM in TU‐OS‐4 cells is 18.2 ± 17.2 μM with progesterone versus 14.8 ± 1.3 μM without progesterone (not significant). The IC₅₀ for GEM in OVKATE cells is 22.4 ± 11.8 μM with progesterone versus 22.4 ± 6.5 μM without progesterone (not significant). Data are presented as the mean and standard deviation (*n* = 3). GEM, gemcitabine; P4, progesterone.

Figure 4 shows the sensitivity of each tumor cell line to PTX. The IC_50_ for PTX in TU‐OS‐4 cells with progesterone was 19.8 ± 6.0 nM, which is 1.8‐fold lower than that in TU‐OS‐4 cells without progesterone (35.3 ± 3.4 nM) (*p* < 0.01). Similarly, the IC_50_ for PTX in OVKATE cells with progesterone was 9.5 ± 32.6 nM, which was 13.5‐fold lower than that of OVKATE cells without progesterone (128.0 ± 64.0 nM) (*p* < 0.01). Conversely, Figure 5 shows no significant difference in the IC_50_ for GEM in TU‐OS‐4 with or without progesterone (18.2 ± 17.2 μM and 14.8 ± 1.3 μM, respectively). Similarly, OVKATE cells showed no significant differences in the IC_50_ for GEM with or without progesterone (22.4 ± 11.8 μM and 22.4 ± 6.5 μM, respectively). Collectively, these results demonstrated that progesterone increased chemosensitivity to PTX, but not to GEM, in ovarian cancer cells.

## 4 Discussion

In this study, we demonstrated that progesterone increased VASH2 expression, enhanced microtubule detyrosination, upregulated cyclin B1 expression, and significantly increased paclitaxel sensitivity in two ovarian cancer cell lines.

We previously showed that progesterone induces cell death in ovarian cancer cells in vitro via non‐genomic mechanisms mediated by mPRs [18]. The ovarian cancer cell lines used in this study are PR‐negative but express mPRs, and the observed effects occurred within 30 min of progesterone exposure. Therefore, the progesterone‐induced effects observed in this study are most likely mediated by non‐genomic signaling through mPRs rather than classical PR‐dependent genomic pathways. However, because the current study did not directly inhibit or knock down mPRs, a definitive mPR‐dependent mechanism could not be established.

VASH2 is a tumor‐specific angiogenic factor, and its inhibition suppresses angiogenesis and tumor progression in various cancers. We have long pursued the development of VASH2‐targeted therapies for ovarian cancer. Previously, shRNA‐mediated knockdown of VASH2 suppressed peritoneal dissemination and prolonged host survival in animal models [7]. Additionally, administration of atelocollagen‐protected siRNA targeting VASH2 markedly inhibited tumor growth in vivo [22]. More recently, we developed a neutralizing anti‐VASH2 antibody that exhibited antitumor efficacy comparable to that of bevacizumab, without apparent adverse effects in ovarian cancer animal models [23].

Recent studies have demonstrated that the VASH family functions as tubulin carboxypeptidases that promote α‐tubulin detyrosination [10, 11]. Consistent with these findings, our previous studies showed that ovarian cancer cells with forced VASH1 expression strongly induced detyrosinated tubulin [24], and CRISPR/Cas9‐mediated VASH2 knockout exhibited reduced detyrosinated microtubules [12]. These observations indicate that VASH1 and VASH2 regulate microtubule dynamics in ovarian cancer cells. In the present study, progesterone treatment was associated with increased VASH2 mRNA expression and enhanced tubulin detyrosination. Although these findings are consistent with increased VASH2‐related activity, we did not directly measure VASH2 protein abundance or enzymatic activity. Therefore, these findings should be interpreted as demonstrating an association rather than direct causality.

Cell cycle progression is tightly regulated by cyclins and cyclin‐dependent kinases (Cdk). Cyclin B1 expression increases from the G2 phase to early M phase, forms a complex with Cdk1, and drives progression through middle M phase (metaphase), after which expression rapidly declines in late M phase (anaphase) [25]. Because balanced tubulin tyrosination and detyrosination are essential for proper microtubule activity, excessive or restrained detyrosination may disrupt microtubule dynamics. The observed increase in cyclin B1, together with enhanced tubulin detyrosination, is consistent with altered microtubule regulation and delayed mitotic progression. However, direct assessment of microtubule dynamics and spindle function was beyond the scope of the present study.

PTX exerts its antitumor effects by binding β‐tubulin, stabilizing microtubules, and inhibiting their dynamic equilibrium, thereby preventing the metaphase‐to‐anaphase transition and inducing apoptosis [26]. Paclitaxel is most effective against cancer cells arrested in the middle M phase (metaphase) [27]. Progesterone pretreatment was associated with significantly reduced paclitaxel IC_50_ values, suggesting that progesterone‐induced molecular alterations may enhance cellular susceptibility to paclitaxel. Conversely, progesterone exposure did not affect sensitivity to GEM, an antimetabolite used to treat recurrent ovarian cancer, possibly because GEM targets DNA rather than microtubules. Additional mechanistic studies are required to determine whether VASH2 is directly responsible for this effect.

Although both VASH1 and VASH2 promote microtubule detyrosination, they exert opposing effects on angiogenesis: VASH1 suppresses angiogenesis, whereas VASH2 promotes it. The anti‐angiogenic effect of VASH1 is mediated by impaired intracellular internalization of vascular endothelial growth factor (VEGF)–VEGF receptor complexes resulting from reduced microtubule activity [28]. Conversely, the mechanism underlying VASH2‐mediated angiogenesis remains unclear. Although the present study did not directly evaluate angiogenesis, identifying VASH2 as a tubulin carboxypeptidase has expanded its biological significance beyond its original role as a pro‐angiogenic factor. Whether VASH2‐mediated regulation of microtubule dynamics and angiogenesis represents independent functions or mechanistically interconnected processes remains unclear. Further studies investigating the relationship between these functions may provide insights into tumor biology and facilitate the development of novel therapeutic strategies targeting the diverse functions of VASH2.

Another limitation is the relatively high progesterone concentrations used in this study. These concentrations exceed physiological circulating progesterone levels and should not be interpreted as reflecting the in vivo endocrine environment. These concentrations were selected based on our previous studies of rapid non‐genomic progesterone responses in ovarian cancer cells, in which comparable experimental conditions reproducibly induced early biological effects without apparent nonspecific cytotoxicity. The primary objective was to investigate early molecular responses to progesterone rather than determine clinically achievable concentrations. Nevertheless, future studies using lower progesterone concentrations, prolonged exposure, and in vivo models are needed to establish the physiological and translational relevance of these findings.

Moreover, the biological effects of progesterone are increasingly recognized as highly context‐dependent and influenced by receptor expression profiles, tumor subtype, and the endocrine microenvironment. Recent evidence suggests that progesterone signaling may exert distinct and occasionally opposing biological effects depending on the cellular context rather than acting uniformly as a protective hormone in ovarian cancer [29]. Therefore, the present findings should not be generalized to all ovarian cancers without careful consideration of tumor heterogeneity and receptor status.

In conclusion, progesterone treatment was associated with increased VASH2 expression, enhanced tubulin detyrosination, and increased paclitaxel sensitivity in PR‐negative ovarian cancer cells. These findings provide in vitro evidence supporting further mechanistic and preclinical investigation of progesterone as a potential adjunct to paclitaxel therapy. Because this study did not establish mPR‐dependent signaling, VASH2 causality, or in vivo efficacy, these findings should be considered hypothesis‐generating.

## Author Contributions

**Takahiro Koyanagi:** conceptualization, investigation, funding acquisition, writing – original draft, methodology. **Yasushi Saga:** conceptualization, investigation, funding acquisition, writing – original draft, methodology. **Yoshifumi Takahashi:** investigation. **Kohei Tamura:** investigation. **Eri Suizu:** methodology, investigation. **Kazuki Yamamoto:** methodology. **Akiyo Taneichi:** methodology. **Yuji Takei:** writing – review and editing. **Hiroaki Mizukami:** writing – review and editing. **Hiroyuki Fujiwara:** supervision.

## Funding

This work was supported by Grant‐in‐Aid for Scientific Research (C) (22K09551) to T.K. and (24K12607) to Y.S. from the Ministry of Education, Culture, Sports, Science, and Technology of Japan.

## Ethics Statement

The authors have nothing to report.

## Conflicts of Interest

The authors declare no conflicts of interest.

## Data Availability

The data that support the findings of this study are available from the corresponding author upon reasonable request.
